# Alternative Methods to Treat Nausea and Vomiting from Cancer Chemotherapy

**DOI:** 10.1155/2015/818759

**Published:** 2015-11-08

**Authors:** Mohammad Ali Sheikhi, Ahmad Ebadi, Abdolhassan Talaeizadeh, Hossein Rahmani

**Affiliations:** ^1^Department of Cardiac Surgery, Atherosclerosis Research Center Golestan Hospital, Ahvaz Jundishapur University of Medical Sciences, Ahvaz, Iran; ^2^Department of Cardiac Anesthesiology, Atherosclerosis Research Center Golestan Hospital and Pain Research Center, Ahvaz Jundishapur University of Medical Sciences, Ahvaz, Iran; ^3^Division of Surgery, Emam Hospital, Ahvaz Jundishapur University of Medical Sciences, Ahvaz, Iran; ^4^Department of Toxicology, Islamic Azad University, Shahreza Branch and Medical Research Center, Jundishapur Health Development Co., Tehran, Iran

## Abstract

Chemotherapy Induced Nausea and Vomiting (CINV) is among the most intensive side effects and critical concerns for patients with cancer. Most of these patients experience nausea and vomiting after chemotherapy. Sometimes, this is so annoying that it may prevent them from continuing the therapy. With the recent advances, a variety of therapeutic methods are innovated and applied to control CINV. Among them, the main methods include medicinal therapy, relaxation, and herbal therapy. Yet, using dexamethasone together with massage therapy and ginger is identified as the most effective method.

## 1. Introduction

CINV is the most serious side effect and the main concern for cancer patients. Its prevalence is reported between 54% and 96% [1]. This side effect induces physiological and electrolytic dysfunctions, changes in immunity system, nutritional disruption, and even esophageal perforation. Consequently, it affects patients' quality of life and the continuance of treatment [2].

Most patients with cancer experience CINV. The increase of excessive vagus excitation due to sympathetic inhibition is among the most important factors in the manifestation of CINV. Many factors increase the chance of CINV. The most important of them include blood pressure drop, the addition of compounds like vascular contractors, neostigmine, and opioids to anesthetics.

Nausea and vomiting are mostly accompanied with sweating, pale skin, and heart palpitation. If not treated, it results in acute dehydration, the imbalance of liquids and electrolytes, and emotional tensions among patients [3]. Similarly, not controlling nausea and vomiting will result in extravagant costs. Among them, direct and indirect costs can be implied. Direct costs include the increase of hospitalization days as well as the extra costs related to medical and nursing cares. Yet, indirect costs include patients', their family members', and (or) caregivers' income loss or reduction. Recently, various methods are applied to control CINV including medicinal or complementary therapies. The selection and prescription of appropriate therapies, medicinal or nonmedicinal therapies, will considerably improve cancer patients' quality of life and performance. They will have desirable effects on their life [4].

With the recent advances, a variety of therapeutic methods are innovated and applied to control CINV. Hence, the present study reviews the methods of reducing CINV in patients with cancer.

## 2. Descried

### 2.1. The Effect of Medicinal Therapy on the Intensity of Nausea and Vomiting in Cancer Patients under Chemotherapy

Among the medicinal methods for the prevention and treatment of CINV, antihistamines, phenothiazines, butyrophenones, and anticholinergics can be implied. Yet, their application is partially limited due to their side effects [5]. Among the antiemetic drugs available, no single antinauseant is capable of controlling CINV on its own. Some of them can be applied in limited dosages due to their side effects. As a result, researchers seek for more effective compounds with fewer side effects.

Dimenhydrinate, midazolam, and metoclopramide are amongst the main drugs prescribed by physicians for controlling and suppressing CINV in cancer patients under chemotherapy.

Dimenhydrinate is an antihistamine drug. It reduces the activity of labyrinth and CTZ center in medulla by its anti-central-muscarinic effect and, finally, hinders nausea and vomiting. Sirin carried out a study concerning the effect of dimenhydrinate on reducing CINV in cancer patients under chemotherapy. He concluded that CINV decreased about 13.8% in patients receiving the drug as compared to the group receiving placebo [6]. In another study, it was also shown that prescribing dimenhydrinate for men with prostate cancer under chemotherapy has reduced their CINV as compared to control group [7]. In their study, Sheanakul et al. reported that the permanent injection of midazolam in cancer patients under chemotherapy has reduced pain and CINV in test group as compared to control group [8]. In a study, it was reported that, hindering histaminic H1 receptor, cinnarizine controls and reduces CINV in cancer patients under chemotherapy as compared to placebo group [9]. In another study, it was reported that metoclopramide reduces CINV in cancer patients under chemotherapy through enhancing the activity threshold of CTZ chemical receptors [10]. In a study by Denehy et al., it was shown that CINV is less in cancer patients under chemotherapy receiving metoclopramide as compared to placebo group [11] (Table 1).

### 2.2. The Effect of Relaxation Methods on the Intensity of Nausea and Vomiting in Cancer Patients under Chemotherapy

Nowadays, nonmedicinal therapies such as relaxation techniques have been proposed for treating CINV.

Some studies show that relaxation response that emerged from massage is effective in controlling nausea [12].

In a study on breast cancer patients receiving radiation therapy, it was reported that patients cited the reduction of pain, uneasiness, nausea, fatigue, and tension, yet the increase of relaxation response and the vital force after massage [13]. Results of a study showed that supportive, behavioral, cognitive, relaxation, and visualization techniques have reduced cancer patients' postchemotherapy pain yet not nausea [14]. In a study by Viswanathan and Eisenberg, it was said that 25 min instruction of relaxation using audio tape was effective in reducing the intensity of delayed nausea and vomiting in patients with cancer [15]. In another study, it was reported that using acupuncture in patients receiving chemotherapy induces antinausea effects. Yet, the results were not statistically significant [16]. Radtke and Lapoint used a combination of music therapy and visualization for reducing anxiety in 15 patients receiving chemotherapy. He found out that anxiety and the intensity of vomiting were significantly reduced. However, no changes were reported regarding the amount of nausea [17]. In a study by Brandt et al., it was reported that moderately massaging patients' back led to relaxation, emotional health, appetite improvement, and nausea reduction in cancer patients receiving chemotherapy [18]. It was shown in a study that ice massage in cancer patients under chemotherapy decreases nausea and vomiting to 45% as compared to control group [19]. Similarly, Chik and Morrow implied the effect of massage in water on reducing CINV in patients with gastric cancer [20].

Based on these results, nurses can effectively apply massage to alleviate pain, nausea in patients with cancer. Accordingly, patients and their families will be trained in this regard. It is noteworthy that one of the main objectives of nursing cares is to provide patients with relaxation and peace. Researcher nurses recommend the application of complementary methods to reduce patients' nausea (Table 2).

### 2.3. The Effect of Adding Medicinal Plants to Conventional Therapy on the Intensity of Nausea and Vomiting in Cancer Patients under Chemotherapy

The wide use of industrial antivomiting drugs for coping with CINV is accompanied with adverse side effects including extra pyramidal side effects, blood pressure drop, and headache. Hence, there has been an increasing tendency to use herbal plants.

Today, plants have drawn considerable interest in the field of treatment as compared to chemical drugs. It is due to having more advantages yet fewer side effects as well as being more cost-effective. Based on a WHO report, about 80% of the world population apply herbal compounds now [21].

Few plans have been used for treating nausea and vomiting, especially, in cancer patients receiving chemotherapy.* Citrus aurantium*,* Hypericum perforatum L.*,* Achillea millefolium L.*, and* Zingiber officinale* are the only plants that induced successful results; especially, in the same regard. They are also of specific significance for researchers.

Orange blossom (scientific name:* Citrus aurantium*) was used in traditional medicine for treating many diseases. During the recent decade, again, it has been considered in controlling CINV. In a study by Zhou et al., it was reported that the application of this plant extract reduces nausea and vomiting in patients with lung cancer as compared to control [22]. In another study, it was shown that nausea and vomiting were reduced in ovarian cancer women receiving* Citrus aurantium* capsules [23].

In the recent decade, we have observed interesting studies regarding the effect of Hypericum (scientific name:* Hypericum perforatum L.*) and Achillea (scientific name:* Achillea millefolium L.*) on the reduction of CINV. For instance, in their study, Laniyonu et al. stated that* Hypericum perforatum L.* reduces CINV in test group as compared to control group [24]. In another study, it was reported that* Achillea millefolium L.* reduces CINV by about 23% in test group as compared to control group [25]. In a study, concerning the effect of* Citrus aurantium*,* Hypericum perforatum L.*,* Achillea millefolium L.*, and* Zingiber officinale* on reducing CINV in various age and gender groups, it was shown that* Zingiber officinale* induces better effect on reducing CINV as compared to other plants mentioned [26].

*Zingiber officinale* is among the herbal plants effective in treating nausea and vomiting without any side effects. In German pharmacopoeia, it is applied to develop antinausea drugs [27]. The major pharmacological activity of ginger (scientific name:* Zingiber officinale*) is related to its active components including gingerol and shogaol. These compounds have antivomiting, antifever, anticoughing, anti-inflammatory, antipressure, and anticancer effects. They also reduce prostaglandin and alleviate digestive problems.* Zingiber officinale* derivatives employ their antivomiting effects through several mechanisms. For instance, gingerol and shogaol decrease gastric contractions yet increase digestive (gastrointestinal) tract activity. These compounds also have antiserotonin effect. They have sweeping effects on free radicals inducing nausea [28]. To examine the application of* Zingiber officinale* for cancer patients, a study was conducted on 50 cancer patients under chemotherapy. The results of this study as “*Zingiber officinale*: An Anti-Vomiting Factor in Postchemotherapy Nausea and Vomiting” showed that* Zingiber officinale* was more effective in controlling nausea and vomiting as compared to metoclopramide [29]. There are studies indicating paradoxical results. For instance, in a study concerning the effect of* Zingiber officinale* on postsurgery nausea and vomiting in 180 women undergoing genital tract laparoscopy, it was shown that this plant has no effect on postsurgery nausea and vomiting. No difference was observed between test and control group [30]. Similarly, a study on 43 cancer patients under chemotherapy aiming to determine the antivomiting effects of* Zingiber officinale* on CINV showed that* Zingiber officinale* is effective in reducing CINV in delayed phase [28].

Regarding the fact that the number of studies emphasizing the effectiveness of* Zingiber officinale* is more than that of the studies rejecting it and concerning study [30] it can be concluded that these results are probably due to the small size of the sample, limited number of participants, and the lack of access to high quality product. This is because the characteristics of the previous studies are so that they can affect their results. In a study, it was implied that 48.3% in* Zingiber officinale* group and 66.7% in placebo group manifested nausea. Hence, the effectiveness of* Zingiber officinale* is 18.4% [31]. The results of another study showed that changes in nausea mean scores were higher among all participants in* Zingiber officinale* group as compared to placebo group. That is, after receiving the diets under study, 28 out of 32 patients in* Zingiber officinale* group (87.5%) and 10 out of 35 patients in placebo group (28.2%) reported nausea improvement [32]. In their study, Levine et al. showed that* Zingiber officinale* together with protein in the diet reduces delayed nausea and the consumption of antinausea drugs [33]. On the other hand, another study indicates the further effectiveness of* Zingiber officinale* as compared to dimenhydrinate with fewer side effects [34]. Similarly, Ozgoli et al. emphasized the application of* Zingiber officinale* as a herbal medication to control CINV in patients receiving chemotherapy [35]. Based on the results of the abovementioned studies, it can be stated that the application of capsules containing* Zingiber officinale* powder can alleviate CINV without any special side effects. Hence, it is possible to improve patients' condition and reduce chemotherapy-induced side effects by providing instructional plans and equipment regarding how to use these capsules (Table 3).

## 3. Conclusion

Based on the studies discussed in this review, it can be said that among the relaxation methods for alleviating CINV, the massage therapy of various members brings about the best results without any side effects depending on the type of cancer. It has also desirable mental effects on patients' health.

Concerning herbal therapies, it can also be reported that researchers in the field of chemotherapy have given prime significance to* Zingiber officinale*. This plant induces acceptable effects on the reduction of CINV.

In the end, based on the results of this study, it can be generally concluded that* Zingiber officinale* (in form of ginger capsule) is the best option among antinausea medications to CINV. Depending on the type of cancer, it is applied due to cost-effectiveness and less side effects as compared to other herbal drugs. During the treatment of CINV, massage therapy is also recommended at certain hours of the day and night due to its desirable effects on reducing patients' nausea and vomiting as well as their mental health.

No studies are available concerning the simultaneous examination of massage therapy, herbal therapy, and dexamethasone therapy regarding the reduction of nausea and vomiting in patients with cancer. Hence, it is recommended that various types of cancers are studied based on abovementioned objective.

In conclusion, herbal and massage therapy is the safety way to treat CINV and it may reduce side effect of toxicity such as liver toxicity.

## Figures and Tables

**Table 1:** The effect of medicinal therapy on the intensity of nausea and vomiting in cancer patients under chemotherapy.

Study	Year	Sample size	Design/population	Method	Outcome
Ekangaki and Chung [5]	2005	450	Cancer patients under chemotherapy in two various age-gender groups	Case-control	The application of histamines, phenothiazines, butyrophenones, and anticholinergics to cope with CINV in chemotherapy brings about side effects
Sirin and Flynn [6]	2013	52	Cancer patients under chemotherapy in two various age-gender groups	Case-control	CINV was reduced by about %13.8 in the group receiving dimenhydrinate as compared to placebo
Pierce and Lin [7]	2013	85	Over 30-year-old men with prostate cancer under chemotherapy	Case-control	Prescribing dimenhydrinate for men with prostate cancer under chemotherapy has reduced their CINV as compared to control group
Sheanakul et al. [8]	2011	30	Below 60-year-old cancer patients under chemotherapy in two gender groups	Case-control	The continuous injection of midazolam has reduced pain and CINV in test group as compared to control group
Apfel et al. [9]	2012	23	15–50 years of age cancer patients under chemotherapy in two gender groups	Case-control	Cinnarizine controls and reduces CINV in cancer patients under chemotherapy as compared to placebo group through hindering histaminic H1 receptor
Rosenthal et al. [10]	2010	64	Over 12-year-old cancer patients under chemotherapy in two various gender groups	Clinical trial	Metoclopramide reduces CINV in cancer patients under chemotherapy through enhancing the activity threshold of CTZ chemical receptors
Denehy et al. [11]	2011	85	Cancer patients under chemotherapy in two various age-gender groups	Case-control	CINV is less in cancer patients under chemotherapy receiving metoclopramide as compared to the group receiving placebo

**Table 2:** The effect of relaxation methods on the intensity of nausea and vomiting in cancer patients under chemotherapy.

Study	Year	Sample size	Design/population	Method	Outcome
Houston and Scholes [12]	2004	75	Cancer patients under chemotherapy in various age-gender groups	Case-control	10 min feet massage is effective in reducing CINV
Lefebvre [13]	2005	120	Over 30-year-old women with breast cancer under radiation therapy	Clinical trial	Massage therapy is effective in reducing nausea
Yusrizal et al. [14]	2006	56	Cancer patients under chemotherapy in various age-gender groups	Case-control	Supportive behavioral, cognitive, relaxation, and visualization techniques did not affect CINV
Viswanathan and Eisenberg [15]	2006	112	Cancer patients with normal hearing under chemotherapy	Case-control	Instruction of relaxation using audio tape was effective in reducing the intensity of delayed nausea and vomiting in patients with cancer
Lapoint et al. [16]	2006	44	Cancer patients under chemotherapy in various age-gender groups	Case-control	Using acupuncture in patients receiving chemotherapy brings about antinausea effects. Yet, results were not statistically significant
Radtke and Lapoint [17]	2004	15	Patients over 12 years of age under chemotherapy in two gender groups	Clinical trial	A combination of music therapy and visualization was not effective regarding the reduction of nausea
Brandt et al. [18]	2005	87	Cancer patients under chemotherapy in various age-gender groups	Case control	Moderately massaging patients' back reduced nausea in cancer patients under chemotherapy
Hummerston et al. [19]	2011	80	Cancer patients under chemotherapy in various age-gender groups	Case-control	Ice massage in cancer patients under chemotherapy decreases the amount of nausea and vomiting to 45% as compared to control group
Chik and Morrow [20]	2013	19	Patients with gastric cancer between 20 and 40 years of age in two gender groups	Case-control	Massaging in water is effective in reducing CINV in patients under chemotherapy

**Table 3:** The effect of adding medicinal plants to conventional therapy on the intensity of nausea and vomiting in cancer patients under chemotherapy.

Study	Year	Sample size	Design/population	Method	Outcome
Zhou et al. [22]	2012	24	Lung cancer patients under chemotherapy in various age-gender groups	Case-control	*Citrus aurantium* extract reduces nausea and vomiting in patients with lung cancer as compared to control group
Pelletier and Ravi [23]	2013	58	Women over 20 years of age with ovary cancer receiving chemotherapy	Case-control	*Citrus aurantium* capsule reduces nausea and vomiting in women with ovary cancer receiving chemotherapy as compared to placebo group
Laniyonu et al. [24]	2011	42	Cancer patients under chemotherapy in various age-gender groups	Case-control	*Hypericum perforatum L.* reduces nausea and vomiting in patients with lung cancer as compared to control group
Tian et al. [25]	2012	70	Patients under chemotherapy in various age-gender groups	Case-control	*Achillea millefolium L.* reduces CINV by about 23% in test group as compared to placebo group
Steingraber and Alrawi [26]	2013	96	Cancer patients under chemotherapy in various age-gender groups	Case-control	*Zingiber officinale* is more effective on reducing CINV as compared to *Citrus aurantium, Hypericum perforatum L.,* and *Achillea millefolium L.*
Eberhart and Sripramote [28]	2011	43	Women with ovary cancer receiving chemotherapy in various age groups	Case-control	*Zingiber officinale* is more effective on reducing CINV in delayed phase
Sontakke et al. [29]	2003	50	Cancer patients under chemotherapy in various age-gender groups	Case-control	*Zingiber officinale* is more effective on reducing CINV as compared to metoclopramide
Eberhart et al. [30]	2003	180	Women undergoing genital tract laparoscopy	Clinical trial	The null effect of *Zingiber officinale* on nausea and vomiting
Nanthakomon and Pongrojpaw [31]	2006	80	Cancer patients under chemotherapy in various age-gender groups	Case-control	The effectiveness of *Zingiber officinale* on nausea and vomiting was reported
Beytz and Ratehanon [32]	2012	67	Cancer patients under chemotherapy in various age-gender groups	Case-control	*Zingiber officinale* is more effective on reducing CINV as compared to placebo
Levine et al. [33]	2008	73	Cancer patients under chemotherapy in various age-gender groups	Case-control	*Zingiber officinale* together with protein in the diet reduces delayed nausea and the consumption of antinausea drugs
Kasper and Pierce [34]	2011	52	Cancer patients under chemotherapy in various ages	Case-control	Further effectiveness of *Zingiber officinale* as compared to dimenhydrinate
Ozgoli et al. [35]	2009	80	Cancer patients under chemotherapy in various ages	Case-control	*Zingiber officinale* is emphasized as a herbal medication for controlling CINV
